# The Spatial Distribution of Local Mobility in Folded Proteins

**DOI:** 10.1021/acs.jpcb.5c07544

**Published:** 2026-01-27

**Authors:** S. Rackovsky

**Affiliations:** Department of Biochemistry and Biophysics, University of Rochester School of Medicine and Dentistry, 601 Elmwood Avenue, Rochester, New York 14642, United States

## Abstract

A quantitative approach is developed to the study of the spatial distribution of amino acid mobility in protein structures. This method, which is based on bioinformatic and signal processing tools, makes it possible to study very large databases of structures simultaneously, and to search for the existence of domains within proteins which are defined by mobility effects, rather than by static structural considerations. It is shown that mobility is distributed nonuniformly in a substantial subset of structures in a large database; that nonuniform mobility distribution does not select for fold class; and that differences in local mobility distribution are correlated with differences in total mobility. Analyzed in light of previous results, these findings suggest that the dynamics of proteins with nonuniform distributions of mobility may exhibit dynamics dominated by local modes, rather than large-scale motions. We suggest that spatial mobility distribution may be a significant driver of protein evolution. It is also speculated that mobility distribution may act as a control on the hydrodynamic environment of proteins in solution.

## Graphical abstract

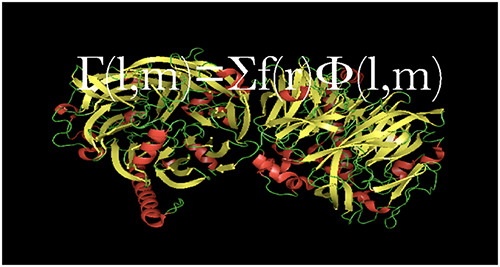

## Introduction

It has long been understood that the mechanisms of folding and function of a protein are critically linked to the dynamic characteristics of the molecule. For this reason, it is of central importance to gain an understanding of the global dynamic organization of proteins. This requires a comprehensive bioinformatic analysis, akin to the many studies which underlie our understanding of the sequences and structures of folded proteins. Until now, however, tools have not been available to carry out such an analysis. Computational studies of protein dynamics have been limited to simulation by molecular dynamics (MD) or by elastic network (EN) models. These approaches, which provide detailed information on atomic length scales for individual molecules, are not suited to the simultaneous analysis of all the proteins in a very large database.

In recent work,^1–7^ we have developed a bioinformatic view of protein dynamics, based on an average, residue-specific α carbon B factor. We denote the average B factor for amino acid X as ⟨B­(X)⟩. (Values of this parameter are given in Table 1.) Any sequence can then be written as a numerical string of ⟨B­(X)⟩ values, in a form which we refer to as the dynamic sequence of the protein. Techniques of signal processing- particularly Fourier analysis- are then applied to the set of dynamic sequences of interest. This representation makes it possible to characterize the mobility distributions of very large groups of structures simultaneously, with extremely modest computational (hardware and runtime) requirements. We have applied this methodology to a unified study of structure and mobility relationships in a large set of folded proteins, and have demonstrated the power of this representation in studying dynamic properties of proteins which are not accessible by any other approach.

**Table 1:** Residue-Specific Average B Factors

	**⟨B⟩**
ALA	26.27
ASP	29.89
CYS	26.27
GLU	31.92
PHE	25.31
GLY	27.45
HIS	26.98
ILE	25.86
LYS	31.30
LEU	26.88
MET	27.65
ASN	28.52
PRO	28.77
GLN	29.54
ARG	29.47
SER	28.32
THR	27.06
VAL	25.67
TRP	23.98
TYR	25.07

In the course of that work, we demonstrated^2^ that the connection between three-dimensional structure and mobility is encoded in the *total* mobility of the sequence, which we denote by β_0_

1 $$\beta_{0}=N^{-1}\sum_{X}\left\langle B\left(X\right)\right\rangle$$

It was shown that β_0_ is correlated with several large-scale properties of proteins:

- β_0_ was shown to act as a controlling variable driving a transition between dynamic regimes in folded proteins, a transition which occurs in differing ways in different folding classes;^3^
- A plausible connection between β_0_ and fold switching was demonstrated;^3^
- The basic determination as to whether a given sequence folds or remains disordered was shown to be controlled by β_0_ and other lowest-*k* Fourier coefficients of the dynamic sequence;^4^
- It was shown that there are significant differences in the distributions of β_0_ between fold classes, and between folded and intrinsically disordered proteins.^6^

In the present work, we begin to study *spatial*, as distinct from *sequential*, aspects of mobility. It is clear that spatially localized mobility must be connected to local flexibility, and therefore to the mechanisms of folding and function of folded proteins. The results embodied herein represent a first step in studying that connection. We have, in fact, demonstrated in previous work^5^ that the choice between folding classes is encoded in intermediate-*k* Fourier coefficients of the dynamic sequence- i.e., by the distribution of mobility along a sequence at intermediate length scales.

We will be concerned herein with several questions concerning the 3-dimensional spatial distribution of mobility, the answers to which are not accessible by conventional approaches:

- To what extent is the mobility of individual residues distributed nonuniformly in the structures of folded proteins?
- If nonuniform distributions of mobility are observed, do they select for particular fold classes?
- Is the three-dimensional distribution of individual amino acid mobility correlated with the total mobility of the protein?

These results will enable us to address a point of fundamental interest, hitherto uninvestigated- the possibility that proteins exhibit domain-like segregation defined by the occurrence of regions with high residue mobility- a property which we refer to as dynamic asymmetry- rather than by purely static structural characteristics. The ability to detect regions of enhanced flexibility- i.e., high localized mobility- in proteins would be an important tool, and has received recent attention^8^ from a simulation point of view. It has been hitherto impossible to search for such domains on a large scale, due to the unsuitability of standard methods for the simultaneous investigation of large data sets of protein structures.

These questions go to the heart of protein behavior. It is reasonable to suppose, for example, that pairs of proteins with similar structures and residue mobility distributions share folding and functional characteristics. It is also reasonable to suppose that pairs of proteins with similar structures but differing distributions of residue mobility fold differently, function differently, or both. With what frequency do such pairs of proteins occur? We have demonstrated, in previous work,^9^ the existence of pairs of proteins which have similar structures and different dynamic behavior. That work was carried out on a small set of preselected proteins. There has been, heretofore, no method available for studying the mobility differences of proteins *en masse*. More particularly, there has been no method available for studying inhomogeneity of mobility within single domains, which are traditionally defined by static geometric properties.

The methods we develop herein lay the groundwork for investigating this and other questions. They represent the first step in an ongoing investigation, the object of which is to connect the rigorously defined spatial distribution of residue mobility to the folding behavior and function of proteins. As a necessary preliminary, a quantitative approach to describing the spatial distribution of amino acid mobility is developed. This approach has the following features:

1. It is based on 3-dimensional, rather than 1-dimensional considerations.
2. We introduce, as a coarse-grained measure of dynamic inhomogeneity, a dynamic dipole. The definition of this directional parameter also makes it possible to consistently define a fixed, dynamics-based coordinate system within any structure.
3. The fine-grained distribution of dynamic characteristics within a given structure is then described, based on that coordinate system, using a spherical harmonic expansion of single-residue < B­(X)> values.
4. The statistical significance of results can be established analytically.
5. Extremely large groups of structures can be analyzed simultaneously.
6. The hardware and runtime requirements of this approach are orders of magnitude less than traditional methods for studying dynamic properties.

The following results are then demonstrated:

- Local mobility is distributed in a statistically significant nonuniform manner in roughly one-quarter of the proteins in a large data set.
- Nonuniform distribution of mobility is not observed to select for fold class.
- Differences in local mobility distribution are, however, correlated with differences in total mobility. The magnitude and sign of this correlation vary by fold class, and these results, together with previous observations, suggest that proteins with regions of dynamic asymmetry tend to be characterized by dynamic modes which are local in character, rather than by those arising from large scale architecture. This observation is in consonance with physical intuition.

## Methods

We require a quantitative measure of the homogeneity, or lack thereof, of the distribution of mobility within a specified structure. We begin by define a characteristic which unites the structural and dynamic properties of a protein in a single quantitative parameter- the mobility dipole. This is calculated as follows:

1. The α carbon center of mass of the structure, **R**_**0**_, is calculated in straightforward fashion.
2. The mobility-weighted C_α_ center of mass, **R**_**β**_, is calculated.
3. From these, the mobility dipole vector **D** = (**R**_**β**_-**R**_**0**_) is calculated.

The mobility dipole vector will be very small for proteins which exhibit a uniform distribution of the residue-dependent mobility ⟨B­(X)⟩ throughout the structure. As the distribution of ⟨B­(X)⟩ becomes less homogeneous, the vector will increase.

The C_α_ center of mass is given by

2 $$R_{0}=\frac{1}{N}\sum_{i=1}^{N}R_{i}$$

and the mobility weighted C_α_ center of mass by

3 $$R_{\beta }=\left[\sum_{i=1}^{N}\beta_{i}R_{i}\right]/\left[\sum_{i=1}^{N}\beta_{i}\right]$$

where **R** *_i_* is the position, and β*_i_* (= ⟨B­(X*_i_*)⟩) is the mobility, of the i*th* α carbon.

The significance of the vector **D** lies in the fact that it defines a direction within the structure determined by the distribution of mobilities. A complete Euclidean coordinate system based on **D** can be constructed from this vector as follows. We first define the unit vector in the *z* direction as

4 $$i_{z}=\frac{D}{\left|D\right|}$$

In order to establish a direction for the unit vector in the x direction, we identify the residue within the structure whose α carbon is farthest from **R**_**0**_. The position vector of that C_α_, in the coordinate system centered on **R**_**0**_, is denoted **R**_**max**_. We then define the unit vector in the *x* direction by the equation

5 $$i_{x}=\frac{\left(R_{\max}\right)\times \left(i_{z}\right)}{\left|R_{\max}\right|}$$

and the unit vector in the *y* direction by

6 $$i_{y}=\left(i_{z}\right)\times \left(i_{x}\right)$$

This coordinate system provides a basis for defining the spatial distribution of mobility within the structure.

We characterize that distribution by performing a spherical harmonic transform, which can be thought of as the 3-dimensional generalization of a Fourier transform. Spherical harmonics are special functions which carry two indices, *l* and *m*, which together describe the angular distribution of the quantity of interest. They define a complete, orthonormal basis over the sphere. Therefore, any real-valued function *f*(**ω**) of spherical variables may be expanded as a linear combination as follows

7 $$f\left(\omega \right)=\sum_{l=0}^{\infty }\sum_{m=-l}^{l}\Gamma \left(l,m\right)\Phi \left(l,m\right)$$

where Φ­(*l*,*m*) is the spherical harmonic with indices *l* and *m*, and the expansion coefficients Γ­(*l*,*m*) can be computed from a projection integral, as

8 Γ(l,m)=∫f(ω)Φ(l,m)dω

In the case of discrete variables, which will be of interest herein, this becomes

9 $$\Gamma \left(l,m\right)=\sum_{r}f\left(r\right)\Phi \left(l,m\right)$$

a form which is particularly convenient because the spherical harmonics Φ­(*l*,*m*) can be expressed in Cartesian form. The first nine harmonics, which will concern us in the present work, are

10 $$\Phi \left(0,0\right)=\sqrt{\frac{1}{4\pi }}$$

11 $$\Phi \left(1,-1\right)=\sqrt{\frac{3}{4\pi }}x$$

12 $$\Phi \left(1,0\right)=\sqrt{\frac{3}{4\pi }}z$$

13 $$\Phi \left(1,1\right)=\sqrt{\frac{3}{4\pi }}y$$

14 $$\Phi \left(2,-2\right)=\sqrt{\frac{15}{4\pi }}\mathrm{xy}$$

15 $$\Phi \left(2,-1\right)=\sqrt{\frac{15}{4\pi }}\mathrm{yz}$$

16 $$\Phi \left(2,0\right)=\sqrt{\frac{5}{16\pi }}\left(3z^{2}-1\right)$$

17 $$\Phi \left(2,1\right)=\sqrt{\frac{15}{8\pi }}\mathrm{xz}$$

18 $$\Phi \left(2,2\right)=\sqrt{\frac{15}{32\pi }}\left(x^{2}-y^{2}\right)$$

The discrete variable of interest in the present case is the mobility of the residue at position **r**, which we denote by β­(**r**). Then the spherical harmonic coefficients become

19 $$\Gamma \left(l,m\right)=\sum_{r}\beta \left(r\right)\Phi \left(l,m;r\right)$$

where we include the spatial variable **r** in the argument of the spherical harmonic to indicate that we use the Cartesian representation.

It is necessary to know the statistical significance of the resulting coefficients. The relevant question is whether these coefficients differ from those which would be obtained from a similar calculation on a randomly permuted version of the original sequence, with the same structure. To this end, we compare the computed spherical harmonic coefficients of the wild-type sequence to the ensemble of coefficients arising from all possible randomly permuted sequences. In previous work^10^ it was shown that the permuted-sequence ensemble averages of coefficients arising from orthonormal expansions of numerically represented sequences, and the associated standard deviations, can be calculated analytically, obviating the need for computationally intensive, and necessarily approximate, estimates resulting from simulations. We adopt this approach in the present work. This makes it possible to represent the coefficients as *Z* functions in the following form

20 $$Z\left(\Gamma \left[l,m\right]\right)=\frac{\Gamma \left[l,m\right]-\left\langle \Gamma \left[l,m\right]\right\rangle }{\sigma \left(\Gamma \left[l,m\right]\right)}$$

where the brackets in the numerator represent the average over all possible sequence permutations.

When the coefficients are represented in this form, their statistical significance is automatically reflected in their numerical values, in standard fashion.

For the present calculations, we use a database of 22278 proteins with known sequence and high-quality structures, with pairwise sequence identity no greater than 60%, derived from the CATH^11^ database. It should be remarked, with regard to computational requirements, that a complete analysis of this database requires approximately 5 min on a MacBook Pro.

## Results and Discussion

We begin by addressing the distribution of mobility in protein structures. It should be noted that, in this work, we consider the properties of single domains. We wish to know the prevalence of statistically significant dynamic asymmetry. Using the measures outlined in the Methods section, and a standard statistical criterion, we define structures with significant dynamic asymmetry as those with at least one spherical harmonic coefficient for which Z­(Γ)≥1.96. We find that 5421 domains- 24.3% of the data set- exhibit significant dynamic asymmetry. In Table 2, we present the populations of the full data set, and of two subsets of the data set, comprising dynamically symmetric and asymmetric domains, for three values of the CATH label C. [It should be remembered that *C* = 1 labels all-helical proteins, C = 2 labels sheet/barrel proteins, and *C* = 3 labels mixed α/β molecules.] We can ask whether the relative populations of the three folding classes differ between the subset of proteins which exhibit significant asymmetry and the complementary, symmetric subset. We address this question by applying a χ^2^ test to the two right-hand columns of the table. We find that there is no significant difference in the relative populations of folding classes between the displaced and undisplaced subsets. There is no selection for asymmetry by fold class.

**Table 2:** Populations of the Database and Subsets by Folding Class

C	all	asymmetic	symmetric
1	5445	1315	4130
2	4973	1210	3763
3	11860	2896	8964

We next ask whether the members of the asymmetric subset differ significantly from dynamically symmetric proteins with respect to total mobility. We find that the average value of β_0_ for the set of proteins with dynamic asymmetry is 27.855, while that for the set of dynamically symmetric proteins is 27.869. This difference, although small, is statistically significant, with *p* = 0.00014. Another way of investigating this question is to determine the degree to which the spherical harmonic coefficients are correlated with β_0_. In Table 3, we show the relevant correlation coefficients. These are all small, but the correlation coefficients for *Z*(1,0) and *Z*(2,0) are noticeably larger in magnitude than others, and negative in sign. It should be noted that, as shown in Figure 1, *Z*(1,0) and *Z*(2,0) are significantly larger in magnitude and/or range than the other coefficients. It will be seen from eqs 12 and 16 that these two coefficients depend only on **i**_*z*_, the direction of the dynamic dipole, and therefore reflect most directly the degree to which asymmetry is present. The negative sign of the correlations corresponds to our observation that the average mobility of the dynamically symmetric subset is larger than that of the subset of asymmetric proteins.

**Figure 1:**
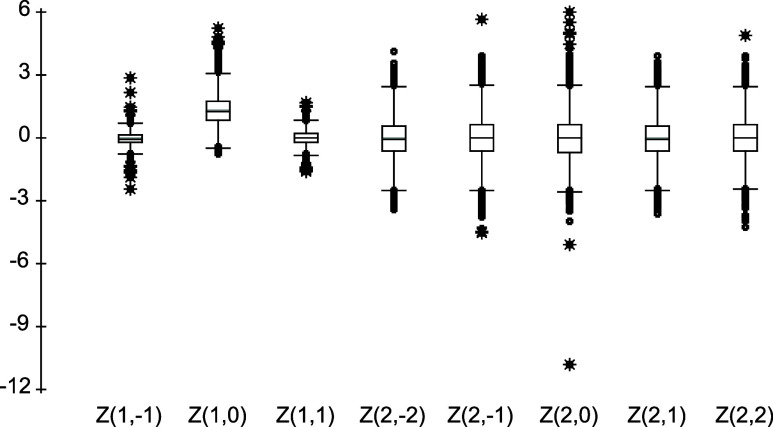
Side-by-side Boxplot of the Distributions of Spherical Harmonic *Z*-functions.

**Table 3:** Correlation of Spherical Harmonic Coefficients With β_0_

*Z*(1,-1)	–0.009
*Z*(1,0)	–0.036
*Z*(1,1)	0.011
*Z*(2,-2)	–0.011
*Z*(2,-1)	0.003
*Z*(2,0)	–0.06
*Z*(2,1)	0.001
*Z*(2,2)	0.01

How does this difference in mobility arise? Is it correlated with fold class? In Table 4 we compare the average values of β_0_ of dynamically symmetric and asymmetric proteins, as a function of the CATH index C. It will be seen that there is a highly significant difference in average β_0_ values between the two groups of helical proteins, but that the difference in average β_0_ values between the two groups of sheet/barrel proteins is not statistically meaningful. The mixed proteins show a smaller, but statistically significant, difference, in the opposite direction to the helical proteins. We have demonstrated in previous work^3^ that, in helical proteins, as β_0_ increases, dynamics are more likely controlled by global structure, rather than local modes, whereas lower β_0_ values are likely associated with dynamics controlled by local modes. It was likewise shown that, in sheet/barrel and α/β proteins, the opposite tendency is observed. These observations suggest that the proteins in the asymmetric group, as a whole, are more likely to exhibit locally controlled dynamics.

**Table 4:** Comparison of Mobilities by Fold Class

C	β_0_ (symmetric)	β_0_ (asymmetric)	*Z*	*p*
1	27.995	27.925	6.42	≪0.001
2	27.815	27.806	1.32	0.19
3	27.833	27.843	–2.44	0.02

## Conclusions

We have demonstrated the following results

- Nonuniform distribution of residue mobility is observed in a large subset of proteins.
- Nonuniform mobility is not correlated with fold class.
- It is, however, correlated with differences in total mobility. The characteristics of this correlation suggest that proteins with regions of dynamic asymmetry are likely to be characterized by dynamic modes which are local in character, rather than by those arising from large scale architecture.

These results indicate that there is indeed high-mobility substructure in a substantial subset of single domains. The connection between that substructure and folding, and various protein functions, such as substrate binding and catalysis, is the subject of ongoing investigation.
